# FLI1 and ERG protein degradation is regulated via Cathepsin B lysosomal pathway in human dermal microvascular endothelial cells

**DOI:** 10.1111/micc.12660

**Published:** 2020-10-09

**Authors:** Celestina Mazzotta, Grace Marden, Alessandra Farina, Andreea Bujor, Marcin A. Trojanowski, Maria Trojanowska

**Affiliations:** ^1^ Arthritis and Autoimmune Diseases Center School of Medicine Boston University Boston MA USA

**Keywords:** Cathepsin B, endothelial cells, ERG, FLI1, scleroderma

## Abstract

**Objectives:**

Friend leukemia integration 1 and erythroblast transformation‐specific, important regulators of endothelial cell homeostasis, are reduced in microvascular endothelial cells in scleroderma patients, and their deficiency has been implicated in disease pathogenesis. The goal of this study was to identify the mechanisms involved in the protein turnover of friend leukemia integration 1 and erythroblast transformation‐specific in microvascular endothelial cells.

**Methods:**

The effects of lysosome and proteosome inhibitors on friend leukemia integration 1 and erythroblast transformation‐specific levels were assessed by Western blotting and capillary morphogenesis. The effect of scleroderma and control sera on the levels of friend leukemia integration 1 and erythroblast transformation‐specific was examined.

**Results:**

The reduction in the protein levels of friend leukemia integration 1 and erythroblast transformation‐specific in response to interferon α or Poly:(IC) was reversed by blocking either lysosomal (leupeptin and Cathepsin B inhibitor) or proteosomal degradation (MG132). MG132, leupeptin or CTSB‐(i) also counteracted the anti‐angiogenic effects of Poly:(IC) or interferon α. Scleroderma sera reduced protein levels of friend leukemia integration 1 and erythroblast transformation‐specific in comparison to control sera. Treatment with CTSB(i) increased the levels of friend leukemia integration 1 and erythroblast transformation‐specific in a majority of serum‐treated samples.

**Conclusions:**

Inhibition of cathepsin B was effective in reversing the reduction of friend leukemia integration 1 and erythroblast transformation‐specific protein levels after treatment with interferon α or scleroderma sera, suggesting that targeting cathepsin B may have a beneficial effect in SSc vascular disease.

**Abbreviations**
CTSB: cathepsin B
dSSc: diffuse systemic sclerosis
ERG: erythroblast transformation‐specific
ETS: E‐twenty‐six
FLI1: friend leukemia integration 1
H sera: healthy sera
IFNα: interferon α
lSSc: limited systemic sclerosis
Leupeptin: diaminomethylidene amino‐1‐oxopentan‐2‐yl‐leucinamide
MG132: N‐benzyloxycarbonyl‐L‐leucyl‐L‐leucyl‐L‐leucinal
MVECs: microvascular endothelial cells
POLY(I:C): polyinosinic‐polycytidylic acid
SSc sera: scleroderma sera

## 1 INTRODUCTION

Friend leukemia integration 1 (FLI1) and erythroblast transformation‐specific (ERG) belong to the E‐twenty‐six (ETS) specific transcription factors family and bind to a consensus DNA sequence centered on the core GGA (A/T) motif through a helix‐loop‐helix domain. Friend leukemia integration 1 has been shown to play a major role in hematopoiesis, embryonic development, and vasculogenesis.^1^ Friend leukemia integration 1 deficiency induced SSc‐like phenotypes in various cell types, including dermal fibroblasts, dermal microvascular endothelial cells (MVECs), and perivascular inflammatory cells.^2^, ^3^, ^4^ It has been proposed, that epigenetic downregulation of FLI1 expression contributes to the profibrogenic phenotype of SSc fibroblasts.^5^ The protein levels of ERG, as well as FLI1, are also reduced in SSc pulmonary vasculature.^6^ Furthermore, endothelial FLI1 deficiency reproduced histopathological and functional abnormalities characteristic of SSc fibrosis and vasculopathy in animal models.^2^, ^7^, ^8^ Unlike FLI1, which is widely expressed, ERG is more specifically expressed in endothelial and hematopoietic cells, where it functions in a manner similar to FLI1.^9^ Erythroblast transformation‐specific and FLI1 have been shown to cooperatively regulate vascular inflammation and EndoMT,^6^, ^10^, ^11^ as well as endoglin gene expression.^12^ Treatment of endothelial cells with proinflammatory stimuli, including LPS, TNF‐α or hypoxia, downregulated ERG expression.^13^ Previous studies have established that FLI1 is primarily regulated at the protein level.^14^, ^15^, ^16^, ^17^ While the ubiquitin‐proteasome pathway has been associated with the turnover of several of the ETS family members, including ERG,^18^, ^19^, ^20^ it is not known whether FLI1 is degraded by this mechanism, particularly in SSc.

The second major protein degradation pathway is lysosomal proteolysis. Lysosomes are ubiquitous organelles which contain approximately 50 soluble hydrolases capable of degrading various macromolecules, including proteins, lipids, and carbohydrates.^21^ Lysosomes perform complex functions including endocytic, phagocytic, and autophagic degradation, antigen presentation, killing of target cells by cytotoxic T‐cells and NK cells, cell adhesion and migration, tumor invasion and metastasis, plasma membrane repair, and protein degradation.^22^, ^23^ Of special interest are the members of the papain family, cysteine proteases cathepsins.^24^, ^25^ Various cathepsins are involved in lysosomal protein recycling and in several physiological processes such as antigen (Ag) processing, wound healing, bone remodeling, prohormone, and proenzyme activation, as well as in pathological conditions including cancer, bronchial asthma, atherosclerosis, periodontitis, rheumatoid arthritis (RA), and osteoarthritis.^26^, ^27^, ^28^ Relevant to our study, it was demonstrated that upregulation of endothelial Cathepsin B enzyme (CTSB) may contribute to the development of SSc vasculopathy, especially to digital ulcers, while reduced expression of CTSB in lesional dermal fibroblasts is likely to be associated with skin sclerosis in early dcSSc.^29^

This study aimed to examine the contribution of the proteasome and lysosome to FLI1 and ERG turnover in MVECs. We showed that both the proteasome and lysosome contribute to the degradation of FLI1 and ERG. Further, we identified CTSB as a key lysosomal enzyme responsible for degradation of FLI1 and ERG.

## 2 MATERIAL AND METHODS

### 2.1 Patients and controls

Serum samples were obtained from patients with limited SSc (n = 2), SSc sine scleroderma^1^ or diffuse SSc (n = 7) (median age 48 years, range 25‐71 years),^30^ and from 10 age‐matched and sex‐matched healthy individuals. Patient characteristics are included in Table 1. Prior to participation, all subjects provided written informed consent according to the Declaration of Helsinki. The study protocol was approved by the Institutional Review Board of Boston University (H‐31479). Peripheral blood samples were collected without any additive, left to clot for 30 minutes before centrifugation at 1500 *g* for 15 minutes, and serum was collected and stored in aliquots at −80°C until used.

**TABLE 1:** Healthy controls and Scleroderma patient's data

ID	Sex	Age	Race	L/D	Organ involvement	Treatment
SSc 1	M	47	white	dcSSc	none	Cellcept
SSc 2	M	71	white	dcSSc	none	Cellcept
SSc 3	F	52	white	dcSSc	none	Methotrexate
SSc 4	F	47	white	ssSSc	PAH	Cellcept
SSc 5	F	53	white	dcSSc	ILD	None
SSc 6	F	31	white	lcSSc	PAH	Prostacyclin
SSc 7	F	54	white	lcSSc	PAH	Prostacyclin
SSc 8	F	47	white	dcSSc	ILD	None
SSc 9	F	66	white	dcSSc	ILD	None
SSc 10	M	62	white	dcSSc	ILD	None
HC 1	M	53	white			
HC 2	M	68	white			
HC 3	M	51	white			
HC 4	M	27	white			
HC 5	F	52	white			
HC 6	M	24	white			
HC 7	F	23	white			
HC 8	F	48	white			
HC 9	F	36	white			

### 2.2 Cell cultures

Microvascular endothelial cells isolated from human foreskin were cultured at 37°C in a humidified atmosphere with 5% CO_2_ on bovine collagen‐coated T25 flasks as previously described^31^, ^32^ in Endothelial Cell Growth Medium supplemented with 5% fetal bovine serum (FBS), 5 ng/mL H‐epidermal growth factor, 1 μg/mL hydrocortisone acetate, 100 U/mL penicillin, 100 μg/mL streptomycin, and 25 μg/mL amphotericin B, without addition of further angiogenic growth factors (MV2 kit, catalog number C‐22121. PromoCell Germany). The culture medium was changed every two days, and after 1 week, a monolayer of primary culture cells with small colonies of polygonal elements was detected, and using immunomagnetic beads recognizing E‐Selectin, they were further identified as MVECs. Following, a second selection with CD31 immunobeads, MVECs were maintained in Endothelial Cell Growth complete Medium and were used between the second and the fourth passages in culture.

In order to find the correct concentration at which the cells result viable and able to control the stress of induction in our experimental conditions, the MVECs were cultured for 24 hours in normal condition or in presence of MG132 1 µM, 0.5 µM, 0.25 µM, 100 nM, 50 µM 10 nM, (Selleckchem Catalog number S2619). We verified that in presence of 100 nM MG132 the layer of cells was adherent to the plates without floating cells dead, and resulting well tolerated by MVECs. In addition, we used 10 Mm of leupeptin, as the minimum concentration suggested by manufacturer in the range of 10‐100 Mm, (Catalog number L2884, SIGMA ALDRICH USA), and 10 ng/mL Cathepsin B inhibitor (CTSB (i)) (Chem Cruz Dallas USA; Catalog number SC‐3131) as which used for VEGF recombinant protein.

### 2.3 Western blot

Microvascular endothelial cells were cultured until 70/75% of confluence. In some experimental conditions, the cells were stimulated with Leupeptin, (10 Μm; Catalog number L2884, SIGMA ALDRICH USA), MG132 (100 nM; Selleckchem Catalog number S2619), Cathepsin B inhibitor (CTSB (i)) (10 ng/mL; Chem Cruz Dallas USA; Catalog number SC‐3131) alone or in combination with interferon α (IFNα; 1000 U for mL; PBL Assay Science; Catalog number 11200‐1 Township, NJ USA). In the initial experiments, we determined working inhibitor concentrations. Microvascular endothelial cells were cultured for 24 hours in the presence of 10 nM, 100 nM, 0.25 µM, 0.5 µM, 1 µM, 50 µM of MG132. We verified that 100 nM MG132 was well tolerated and did not affect cell viability. We used 10 mM of leupeptin, as the minimum concentration suggested by the manufacturer is in the range of 10‐100 mM. The concentration of Cathepsin B inhibitor (10 ng/mL) was chosen to be in the range of the levels of Cathepsin B in human serum (10‐65 ng/mL).^29^ In some experiments, cells were treated with 5% of Healthy or scleroderma sera (SSc) sera alone or in combination with 10 ng/mL Cathepsin B inhibitor, then scraped in order to prepare a dry pellet and extract the total protein using lysis buffer with the following composition: 1% Triton X‐100, 50 mmol/L Tris‐HCl (pH 7.4), 150 mmol/L NaCl, 3 mmol/L MgCl2, 1 mmol/L CaCl2, proteinase inhibitor mixture (Roche), and 1 mmol/L phenylmethyl sulfonyl fluoride. Fifteen micrograms of total proteins were electrophoresed using SDS‐PAGE and blotted to nitrocellulose membranes. The membranes were blocked for two hours with 2.5% milk and incubated overnight at 4°C with the following primary Ab_s_: mouse monoclonal anti‐human FLI1 (1:750 dilution, BD Biosciences, Billerica, MA), rabbit monoclonal anti‐human ERG (1:2000 dilution, Cell Signaling, USA), and mouse monoclonal anti‐human β‐actin (1:5000 dilution; Sigma, St Louis, MO), washed, and incubated on a rotary shaker at room temperature (RT), for 1 hour with appropriate HRP‐conjugated secondary Ab (anti‐rabbit LNA 934V/AH, or anti‐mouse LNA931V/AH, GE Healthcare UK). After washing, immunodetection was performed by ECL (Pierce, Rockford, IL). Protein levels were quantified using Image J software, and the values were normalized to β‐actin.

### 2.4 In vitro capillary morphogenesis

In vitro capillary morphogenesis was performed in 96‐well plates covered with 50 μL of Matrigel (CORNING Bedford, MA, USA). The Matrigel was inserted into culture wells, and polymerized for 30 minutes at 37°C; MVECs (30 × 10^3^ cells/well) were incubated in Endothelial Cell Growth complete medium with MG132 (100 nM), Leupeptin (10 μM), CTSB (i) (10 nM), Poly:(IC) (1 μM), IFNα (1000 U/mL) alone or in combination with MG132 (100 nM) + Poly:(IC) (1 μM), MG132 (100 nM) + IFNα (1000 U/mL), Leupeptin (10 μM) + Poly:(IC) (1 μM), Leupeptin (10 μM) + IFNα (1000 U/mL), CTSB (i) (10 nM) + Poly:(IC) (1 μM), and CTSB (i) (10 nM) + IFNα (1000 U/mL). The wells were photographed at 24 hours. The results were quantified by measuring the percent field occupancy of capillary projections, as determined by image analysis. Six to nine photographic fields from three plates were scanned for each experimental point. A *P* value <.05 was considered significant.

### 2.5 Statistical analysis

Data are expressed as the mean ± SEM. ANOVA and Tukey's correction multiple comparisons or Student's *t* test were used where appropriate for statistical evaluation of the differences between independent groups. A *P* value <.05 was considered statistically significant.

## 3 RESULTS

### 3.1 Protein turnover of FLI1 and ERG in MVECs is mediated by the proteasome and lysosome

**FIGURE 1:**
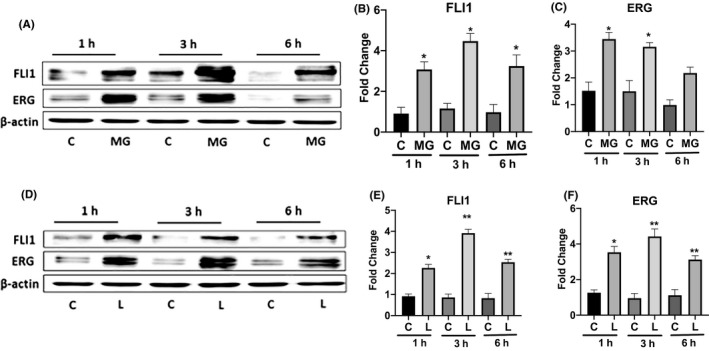
Treatment with MG132 or Leupeptin increases protein levels of FLI1 and ERG in MVECs. A, Western Blot of total proteins extracted from MVECs treated with MG132 for 1, 3, and 6 hours and assayed with anti‐FLI1 Ab and anti‐ERG Ab. D, Western Blot of total protein extracted from MVECs treated with Leupeptin, for 1, 3, and 6 hours and assayed with anti‐FLI1 Ab and anti‐ERG Ab. Representative immunoblots are shown. B, C, E, and F, densitometric analysis of the bands normalized to β‐actin. Results are representative of five independent experiment performed with five different cell lines. Data are mean ± SEM of optical density (OD) in arbitrary units. Student's *t* test was used for statistical analysis

**FIGURE 2:**
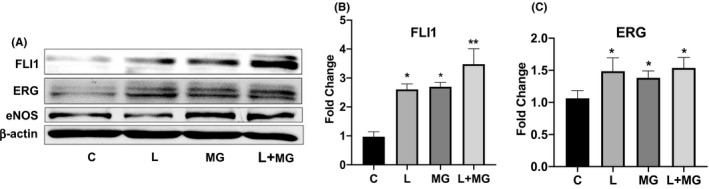
MG123 and Leupeptin have comparable effects on FLI1 and ERG protein levels. A, Western Blot of total protein extracted from MVECs treated for 24 hours with MG132, Leupeptin added alone or together, and tested with anti‐FLI1 Ab, anti‐ERG Ab, and eNOS Ab. Representative immunoblots are shown. The densitometric analysis of the bands normalized to β‐actin. B, C, densitometric analysis of the bands normalized to β‐actin. Results are representative of five independent experiment performed with five different cell lines. Data are mean ± SEM of optical density (OD) in arbitrary units. Student's *t* test was used for statistical analysis

In order to determine whether the proteasome or lysosome are involved in the steady‐state protein turnover of FLI1 and ERG, MVECs were treated with commonly used proteasome (MG132) or lysosome (Leupeptin) inhibitors. As displayed in Figure 1A, treatment with 100 nM of MG132 for 1, 3, and 6 hours, significantly increased FLI1 protein levels compared with each control, with a maximal increase at 3 hours (Figure 1B), and the increased protein levels were sustained up to 24 hours (Figure 2A,B). Under the same experimental conditions, maximal increase of ERG protein levels occurred at 1 hour and persisted for 3 hours, with a smaller increase at 6 hours (Figure 1A,C) which was maintained up to 24 hours (Figure 2A,B). Likewise, inhibition of the lysosomal pathway by leupeptin (10 μM) for 1, 3, 6, and 24 hours showed a significant increase of FLI1 protein compared with each control (Figures 1D,E and 2A,B). Treatment with leupeptin also resulted in increased levels of ERG protein (Figure 1D,F), which extended for up to 24 hours (Figure 2A,C). Inhibition of lysosome or proteasome degradation had comparable effects on the levels of FLI1 and ERG proteins (Figure 2A,B). Simultaneous blockade of lysosome and proteasome was comparable to the addition of each inhibitor alone. Since previous work has shown that eNOS degradation is regulated by the proteosomal pathway in bovine pulmonary artery endothelial cells,^33^ we also assessed the protein levels of eNOS in our experimental system. We confirmed upregulation of eNOS protein levels in MVECs treated with MG132; however, treatment with leupeptin did not affect eNOS levels (Figure 2A). Taken together, these data demonstrate that both, lysosome and proteasome, are involved in degradation of FLI1 and ERG proteins in MVECs.

### 3.2 CTSB regulates lysosomal degradation of FLI1 and ERG proteins

**FIGURE 3:**
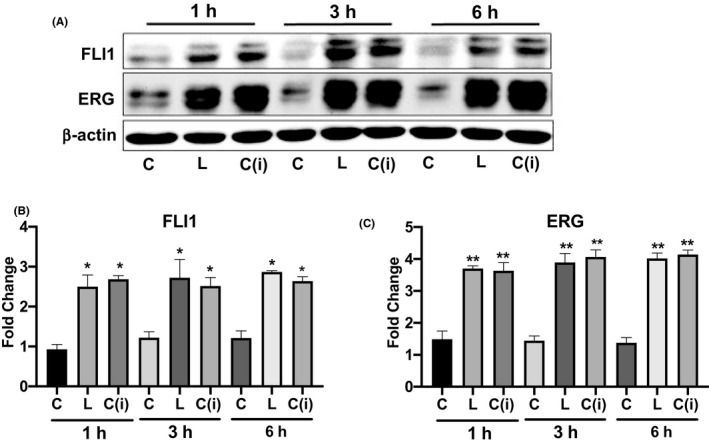
Cathepsin B mediates degradation of FLI1 and ERG in MVECs. A, Western Blot of total proteins extracted from MVECs treated with Leupeptin or Cathepsin B inhibitor for 1, 3, and 6 hours and assayed with anti‐FLI1 Ab and anti‐ERG Ab. Representative immunoblots are shown. B and C, The densitometric analysis of the bands was normalized to β‐actin. The results are representative of five independent experiment using five different cell lines. Data are mean ± SEM of optical density (OD) in arbitrary units. Student's *t* test was used for statistical analysis

Considering that leupeptin is capable of inhibiting several lysosomal enzymes, and the levels of CTSB were increased in dermal blood vessels in vivo in SSc skin,^29^ we focused on CTSB, a ubiquitous hydrolase produced by different cell types, including endothelial cells. The effects of leupeptin and cathepsin B inhibitor (CTSB‐i) on the protein levels of FLI1 and ERG were compared side by side in MVECs treated with each compound for 1, 3, and 6 hours. As shown in Figure 3A, inhibition of cathepsin B, produced a significant increase of FLI1 and ERG protein levels, that was comparable to that of leupeptin at all time points tested (Figure 3B,C). These data demonstrate that cathepsin B is a primary lysosomal enzyme responsible for degradation of FLI1 and ERG in MVECs.

### 3.3 Inhibition of the proteasome and lysosome reverses IFNα‐mediated FLI1 and ERG downregulation

**FIGURE 4:**
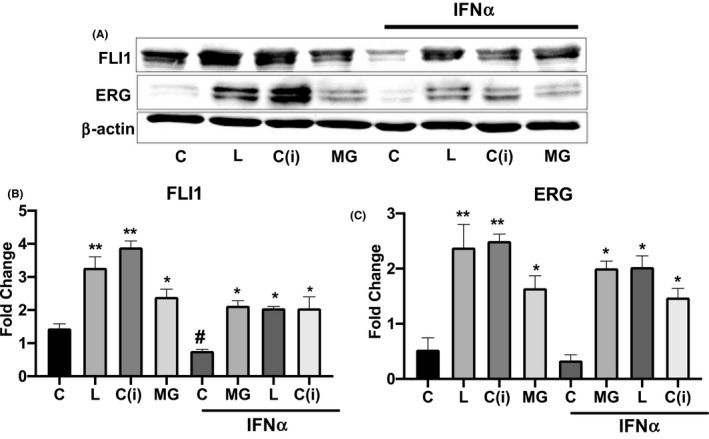
Effect of Leupeptin, Cathepsin B inhibitor, and MG132 on the protein levels of FLI1 and ERG in MVECs treated with IFNα. A, Western Blot of total proteins extracted from MVECs treated with Leupeptin, Cathepsin B inhibitor, or MG132 with or without addition of IFNα. Representative immunoblots are shown. B and C, The densitometric analysis of the bands was normalized to β‐actin. The results are representative of three independent experiment using five different cell lines. Data are mean ± SEM of optical density (OD) in arbitrary units. Student's *t* test was used for statistical analysis

Activation of type I interferons plays a key role in SSc pathogenesis.^34^, ^35^, ^36^ Furthermore, IFNα and IFNβ are potent inhibitors of angiogenesis^37^, ^38^, ^39^, ^40^ and have been implicated in the impairment of endothelial cells in SSc.^41^, ^42^ To determine if leupeptin, cathepsin B inhibitor, or MG132 could counteract the negative effect mediated by IFNα on FLI1 and ERG expression, MVECs were treated with MG132, leupeptin, or cathepsin B inhibitor in the presence or absence of IFNα for 24 hours (Figure 4A). Consistent with previous reports, addition of IFNα reduced FLI1 and ERG protein levels compared with control (Figure 4). Treatment with MG132, leupeptin or cathepsin B inhibitor mitigated the inhibitory effect of IFNα (Figure 4B,C). Interestingly, while treatment with MG132 resulted in comparable levels of FLI1 and ERG in IFNα treated and untreated cells, treatment with leupeptin or cathepsin B inhibitor only partially restored the FLI1 and ERG levels in the IFNα treated cells (Figure 4B,C). This may suggest that in the presence of IFNα endothelial cells primarily use the proteosomal pathway to degrade FLI1 and ERG. However, additional studies are needed to better understand the effects of IFNα on FLI1/ERG protein turnover in MVECs.

### 3.4 Inhibition of the proteasome or lysosome restores capillary morphogenesis in MVECs

**FIGURE 5:**
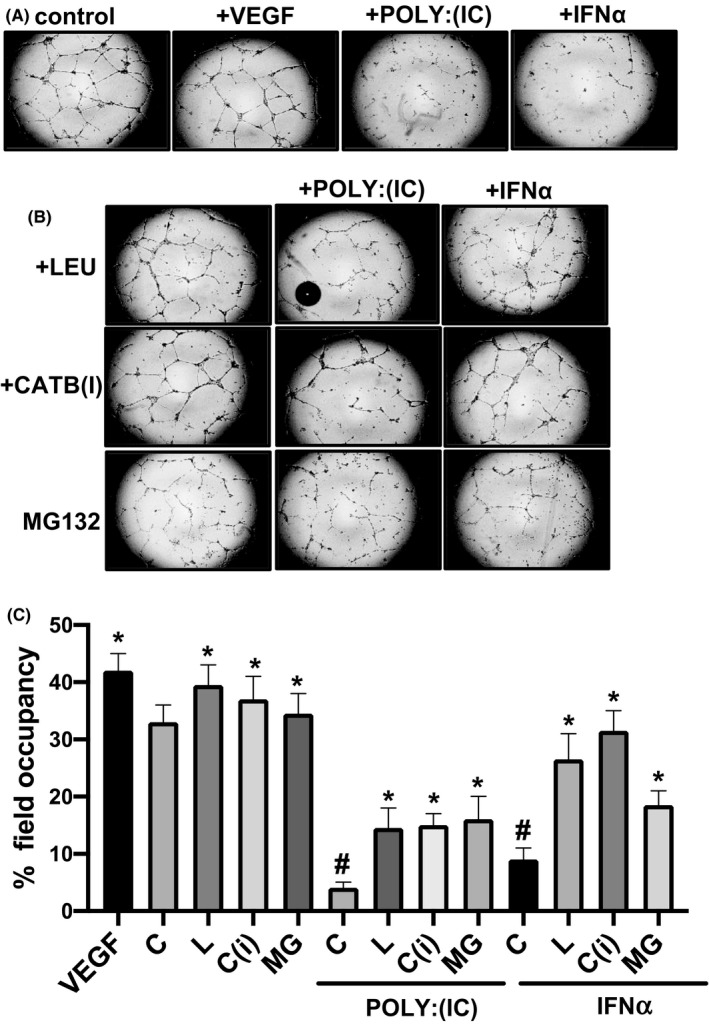
Effect of Leupeptin, Cathepsin B inhibitor, and MG132 on capillary morphogenesis. A and B, representative images of the capillary network formed on Matrigel in MVECs treated with Leupeptin, Cathepsin B inhibitor, or MG132 with or without addition of poly(I:C) or IFNα. Treatment with vascular endothelial growth factor (VEGF) was included as a positive control for angiogenesis. C, quantitative analysis of capillary morphogenesis as percent field occupancy of capillary projections at 24 hours. Data are means ± SEM of three independent experiments performed in triplicates with each one of the three cell lines. ANOVA *t* test with Tukey's correction for multiple comparisons was used for statistical analysis; *P* values are indicated

To investigate the functional influence mediated by the proteasome and lysosome pathways on in vitro angiogenesis, we employed a Matrigel capillary morphogenesis assay. In this assay, endothelial cells are able to create elongated processes, forming anastomosing cords of cells similar to a tubular capillary plexus. Microvascular endothelial cells were treated for 24 hours with Poly:(IC) or IFNα in combination with leupeptin, MG132, or cathepsin B inhibitor. Treatment with Poly:(IC) and to a lesser extent with IFNα resulted in a significant reduction of tube formation in Matrigel (Figure 5A). Treatments with leupeptin, cathepsin B inhibitor, and MG132 partially restored tube formation in Poly:(IC) treated cells with all three treatments showing a comparable effect (Figure 5B,C). In IFNα treated cells, the ability to form tubes was almost fully restored by leupeptin and cathepsin B inhibitor, while treatment with MG132 was somewhat less effective (Figure 5B,C). Together, these data suggest that the anti‐angiogenic pathways activated by IFNα could be modulated through the regulation of the proteasome or lysosomal activity, and in particular by regulating the action of CTSB enzyme.

### 3.5 CTSB inhibition reverses the inhibitory effect of SSc patient sera on FLI1 and ERG protein levels

**FIGURE 6:**
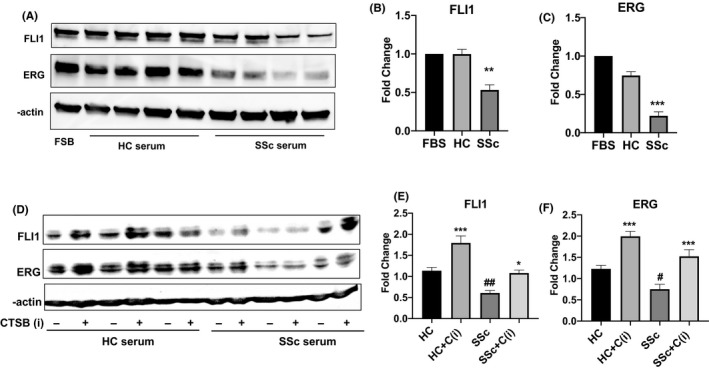
Cathepsin B inhibition reverses the inhibitory effect of SSc patient sera on FLI1 and ERG protein levels. A, Western Blot of total proteins extracted from MVECs treated with HC or SSc sera for 72 hours and assayed with anti‐FLI1 Ab and anti‐ERG Ab. B and C, The densitometric analysis of the bands was normalized to β‐actin. Data are mean ± SEM of optical density (OD) in arbitrary units. Student's *t* test was used for statistical analysis. D, Western Blot of total proteins extracted from MVECs treated with HC or SSc sera alone or in combination with CTSB inhibitor for 24 hours and assayed with anti‐FLI1 Ab and anti‐ERG Ab. E and F, The densitometric analysis of the bands normalized to β‐actin. Data are mean ± SEM of optical density (OD) in arbitrary units, based on 7 SSc and 7 HC sera. Student's *t* test was used for statistical analysis

Previous studies have shown that treatment with SSc serum reduced FLI1 protein levels in MVECs.^14^, ^15^ We confirmed these findings in our study (Figure 6A,B). We also showed that similar to FLI1, the levels of ERG were reduced in MVECs upon the treatment with SSc sera (Figure 6A,C). Given that circulating levels of cathepsin B are elevated in SSc sera,^29^ we asked whether Cathepsin B inhibition would restore the levels of FLI1 and ERG protein in serum‐treated MVECs. Microvascular endothelial cells were treated for 24 hours with healthy or SSc sera alone or in combination with cathepsin B inhibitor. Addition of cathepsin B inhibitor increased FLI1 and ERG levels in a majority of cells treated with healthy serum. However, CTSB inhibitor had a variable effect on the levels of FLI1 and ERG in cells treated with SSc serum with ERG being somewhat more responsive to the treatment than FLI1 (Figures 6D‐F and S1). Together, these data suggest that the modulation of CTSB activity could partially ameliorate the harmful effects of SSc sera on endothelial cells.

## 4 DISCUSSION

Endothelial cell dysfunction contributes to the development of a broad spectrum of diseases, including cancer, diabetes, arteriosclerosis, and autoimmune diseases.^43^, ^44^ Endothelial cells play a crucial role in inflammatory processes by maintaining vascular integrity and immune cell trafficking. Vascular inflammation and impaired angiogenesis are the early pathogenic events in SSc leading to capillary drop‐out and excessive vascular remodeling.^45^ Because dysregulation of the FLI1 and ERG signaling pathways contribute to the development of vascular disease in SSc,^7^, ^46^ this study focused on delineating the molecular mechanisms involved in the regulation of FLI1 and ERG degradation under inflammatory conditions represented by SSc sera and IFNα. The choice of IFNα was based on the well‐documented high levels of IFNα in the blood and skin of SSc patients and its prominent pathogenic role in SSc.^34^, ^35^, ^36^ Here, we showed that both the proteasome and lysosome are involved in the turnover of FLI1 and ERG proteins in MVECs. Based on the previous report that implicated lysosomal enzyme cathepsin B as a potential contributor to the development of SSc vasculopathy, we focused on its role in FLI1 and ERG protein degradation.^29^ We found that cathepsin B plays a key role in maintaining steady‐state protein levels of both FLI1 and ERG and its inhibition resulted in a significant increase of FLI1 and ERG proteins in MVECs.

Under pathological conditions such as rheumatoid arthritis or in certain cancers, cathepsin B can be excessively secreted by different cell types leading to degradation of the extracellular matrix (ECM), activation of other proteases, and liberation of cytokines from the ECM.^47^, ^48^, ^49^ In RA, elevated expression of CTSB in synovial fibroblasts located at the sites of cartilage and bone erosion was shown to significantly contribute to their invasive phenotype.^50^, ^51^ Likewise, elevated levels of CTSB alone or in combination with members of other proteolytic pathways have been linked to tumor progression.^52^ Interestingly, cathepsin B has also been shown to regulate post‐translational processing of tumor necrosis factor‐α (TNF‐α) by facilitating TNF‐α containing vesicle trafficking to the plasma membrane.^53^ A recent study by Noda et al demonstrated that SSc fibroblasts express lower levels of CTSB, which could be reversed by blocking autocrine TGFβ signaling.^29^ On the other hand, the levels of CTSB were increased in dermal blood vessels in vivo in SSc skin,^29^ suggesting that in SSc patients elevated CTSB could be involved in defective neo‐angiogenesis by increasing the turnover of FLI1 and ERG protein levels. Of note, it has been reported that in bovine retinal ECs excessive CTSB interferes with the angiogenic process by downregulating VEGF and upregulating angiogenesis inhibitor endostatin.^54^ Consistent with the latter study, we have shown that inhibition of CTSB restored the ability to form tubes in MVECs treated with IFNα in the in vitro capillary morphogenesis assay.

Published studies and our new data indicate that factors present in SSc sera reduce FLI1 protein levels in MVECs. Here, we showed for the first time that ERG protein is also downregulated by SSc sera. FLI1 and ERG could be regulated by a variety of inflammatory mediators; therefore, the specific factor(s) responsible for downregulation of FLI1 and ERG in patient sera used in our experiments are not known. Since ERG and FLI1 play a central role in maintaining endothelial cell homeostasis by regulating the key cellular processes, their concurrent downregulation is likely to have a significant harmful impact on the vasculature of SSc patients, including impaired neo‐angiogenesis, thrombosis, and endothelial to mesenchymal transition.^2^, ^16^, ^55^, ^56^, ^57^ Accordingly, restoration of the endothelial levels of FLI1 and ERG in SSc patients would be helpful in ameliorating vascular disease in SSc patients. In this work, we showed that inhibition of CTSB has the ability to reverse the harmful effects of inflammatory mediators present in SSc sera on Fli1 and ERG protein levels, suggesting that targeting cathepsin B could be an attractive strategy for SSc vascular disease. Because of the widespread association of cathepsin B with serious diseases such as cancer, autoimmune, and neurological diseases, there is a great interest in developing potent and selective CTSB inhibitors for clinical use.^58^, ^59^ However, because of the complex role of cathepsin B in normal physiological processes, broad‐spectrum inhibition of cathepsin B may lead to undesired off‐target effects. Thus, the development of novel highly selective and reversible CTSB inhibitors is needed before such compounds could be safely used in clinic. If such drugs would become available, they could be very helpful for treatment of vascular diseases associated with ERG and Fli1 deficiency.

## 5 PERSPECTIVES

We report that lysosomal enzyme cathepsin B plays a central role in regulating FLI1 and ERG turnover in MVECs. Further, we showed that SSc sera downregulate FLI1 and ERG proteins and that the inhibition of cathepsin B has the ability to reverse these effects. Targeting cathepsin B could represent an attractive strategy for vascular disease in SSc.

## AUTHORS' CONTRIBUTIONS

C. Mazzotta: conceived the study, participated in study design and coordination, contributed to most of the experiments, analysis, and interpretation of data, drafted and edited the manuscript, and gave final approval. G. Marden: performed some of the experiments, contributed to data acquisition and analysis, and gave final approval. GA Farina: collected and supplied biological samples and clinical data, contributed to analysis of data, and gave final approval. Marcin A. Trojanowski: collected and supplied biological samples and clinical data, contributed to analysis of data, and gave final approval. A. Bujor: collected and supplied biological samples and clinical data, contributed to analysis and data interpretation, and gave final approval. Maria Trojanowska: contributed to conception and design of the study, data analysis, and interpretation, drafted the manuscript, and gave final approval.

## Supporting information

Fig S1

## Data Availability

The raw data supporting the conclusions of this manuscript will be made available by the authors, without undue reservation, to any qualified researcher.
