# Supplementary figures and images for: FLI1 and ERG protein degradation is regulated via Cathepsin B lysosomal pathway in human dermal microvascular endothelial cells
Source: Microcirculation. 2020 Oct 9;28(1):e12660. doi: 10.1111/micc.12660 (PMC7988617; doi:10.1111/micc.12660)

## Slide 1
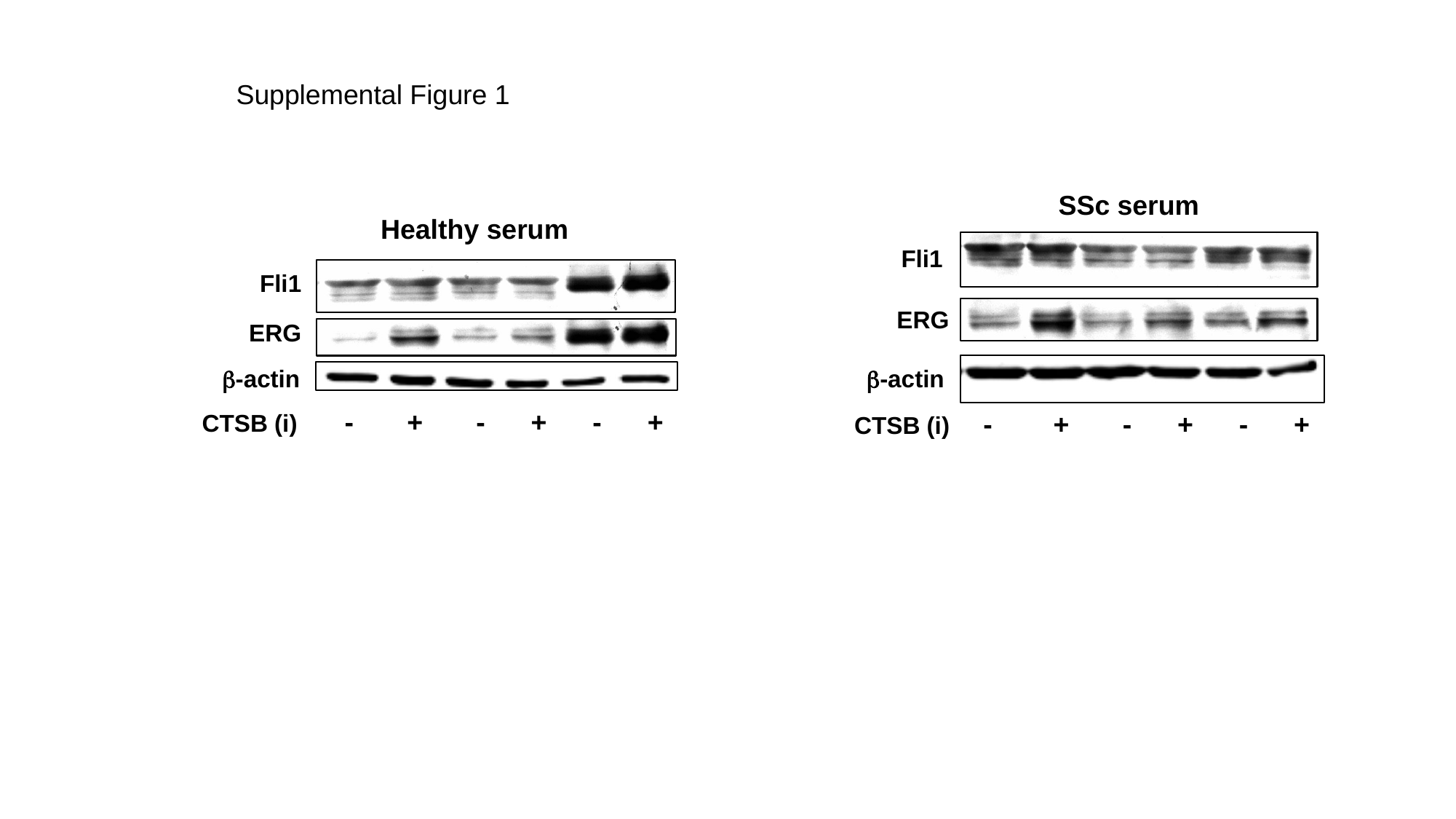

Supplemental Figure 1
SSc serum
Healthy serum
Fli1
Fli1
ERG
ERG
b-actin
b-actin
CTSB (i) - + - + - +
CTSB (i) - + - + - +

Supplement: Supplementary file 1 — Fig S1 [file MICC-28-e12660-s001.pptx]
